# P-109. Building a Rapid Clinical Research Network for Infectious Disease Emergencies in Japan: The iCROWN Initiative

**DOI:** 10.1093/ofid/ofaf695.337

**Published:** 2026-01-11

**Authors:** Kento Kimpara, Hiromi Hibino, Tetsuya Fukuda, Masahiro Kazama, Norio Ohmagari, Daisuke Tokita, Hiroyuki Noda, Takanori Funaki, Teiji Takei

**Affiliations:** Japan Institute for Health Security, Shinjuku, Tokyo, Japan; Japan Institute for Health Security, Shinjuku, Tokyo, Japan; Japan Institute for Health Security, Shinjuku, Tokyo, Japan; Japan Institute for Health Security, Shinjuku, Tokyo, Japan; Japan Institute for Health Security, Shinjuku, Tokyo, Japan; Japan Institute for Health Security, Shinjuku, Tokyo, Japan; Japan Institute for Health Security, Shinjuku, Tokyo, Japan; Japan Institute for Health Security, Shinjuku, Tokyo, Japan; Japan Institute for Health Security, Shinjuku, Tokyo, Japan

## Abstract

**Background:**

Japan’s initial COVID-19 response faced challenges due to insufficient collaboration between industry, government, and academia, a limited number of hospitals prepared for clinical trials, and the absence of national systems for data and specimen sharing. These shortcomings hindered progress in developing domestic vaccines and therapeutics. In fiscal year 2021, the Ministry of Health, Labour and Welfare launched the Repository of Data and Biospecimen of Infectious Diseases (REBIND), a secure platform for collecting clinical data and biospecimens and distributing them to qualified researchers. In fiscal year 2024, REBIND was expanded into the Infectious-disease Clinical Research Network (iCROWN).

**Methods:**

iCROWN currently includes approximately 30 designated medical institutions across Japan. It is overseen by the Japan Institute for Health Security, which manages an administrative board and six expert committees. In addition to REBIND’s original functions, iCROWN operates a clinical research platform and conducts regular preparedness training to enable swift emergency response. Standardized case-report forms, material transfer agreements, and preapproved protocols allow any site to launch a study with a single signature. Engagement is sustained through advisory boards, councils, town hall meetings, and skills-based workshops.

**Results:**

Ongoing collaboration has established a robust platform capable of responding to emerging pathogens. In fiscal year 2024, one multicenter investigator-initiated trial and one clinical study on Severe Acute Respiratory Infection (SARI) were launched. Regular tabletop exercises assess surge capacity and improve operational processes.

**Conclusion:**

iCROWN is expected to play a role in collecting and sharing data to accurately assess infectious disease emergencies and support effective response strategies. It also aims to accelerate clinical research and streamline regulatory processes for medical countermeasures.

Note: This abstract was reviewed using Grammarly.

**Disclosures:**

All Authors: No reported disclosures

